# The Role of Vascular Endothelial Growth Factor Receptor-1 Signaling in the Recovery from Ischemia

**DOI:** 10.1371/journal.pone.0131445

**Published:** 2015-07-02

**Authors:** Hideki Amano, Shintaro Kato, Yoshiya Ito, Koji Eshima, Fumihiro Ogawa, Ryo Takahashi, Kazuki Sekiguchi, Hideaki Tamaki, Hiroyuki Sakagami, Masabumi Shibuya, Masataka Majima

**Affiliations:** 1 Departments of Pharmacology, Kitasato University School of Medicine, Kanagawa, Japan; 2 Departments of Surgery, Kitasato University School of Medicine, Kanagawa, Japan; 3 Departments of Immunology, Kitasato University School of Medicine, Kanagawa, Japan; 4 Departments of Anatomy, Kitasato University School of Medicine, Kanagawa, Japan; 5 Gakubunkan Institute of Physiology and Medicine, Jobu University, Gunma, Japan; University of Illinois at Chicago, UNITED STATES

## Abstract

Vascular endothelial growth factor (VEGF) is one of the most potent angiogenesis stimulators. VEGF binds to VEGF receptor 1 (VEGFR1), inducing angiogenesis through the receptor’s tyrosine kinase domain (TK), but the mechanism is not well understood. We investigated the role of VEGFR1 tyrosine kinase signaling in angiogenesis using the ischemic hind limb model. Relative to control mice, blood flow recovery was significantly impaired in mice treated with VEGFA-neutralizing antibody. VEGFR1 tyrosine kinase knockout mice (TK^-/-^) had delayed blood flow recovery from ischemia and impaired angiogenesis, and this phenotype was unaffected by treatment with a VEGFR2 inhibitor. Compared to wild type mice (WT), TK^-/-^ mice had no change in the plasma level of VEGF, but the plasma levels of stromal-derived cell factor 1 (SDF-1) and stem cell factor, as well as the bone marrow (BM) level of pro-matrix metalloproteinase-9 (pro-MMP-9), were significantly reduced. The recruitment of cells expressing VEGFR1 and C-X-C chemokine receptor type 4 (CXCR4) into peripheral blood and ischemic muscles was also suppressed. Furthermore, WT transplanted with TK^-/-^ BM significantly impaired blood flow recovery more than WT transplanted with WT BM. These results suggest that VEGFR1-TK signaling facilitates angiogenesis by recruiting CXCR4^+^VEGFR1^+^ cells from BM.

## Introduction

Angiogenesis is a complex and tightly regulated process that forms new blood microvessels [1]. Vascular endothelial growth factor (VEGF) is one of the most potent angiogenic stimulators [2]. The VEGF pathway plays a critical role in ischemic angiogenesis and tumor growth through diverse mechanisms [2, 3, 4]. VEGFA binds to two receptors tyrosine kinases, VEGF receptor 1 (VEGFR1) and VEGF receptor 2 (VEGFR2). VEGFR2 is expressed mainly in endothelial cells. VEGFR1 is expressed not only in endothelial cells, but also in hematopoietic stem cells and inflammatory cells, such as monocytes and macrophages, in which it regulates chemotaxis [5,6,7]. VEGFR1 binds VEGFA with an affinity approximately 10 times higher than that of VEGFR2, but its precise biological mechanism is not fully understood. VEGFR2-null mice fail to develop blood vessels and die *in utero*, indicating that VEGFR2 signaling is essential for the development of the vascular system [8]. By contrast, VEGFR1-null mice exhibit overgrowth and disorganization of blood vessels, which suggests that VEGFR1 is a negative regulator of angiogenesis during embryonic development. However, transgenic mice expressing a variant of VEGFR1 that lacks the tyrosine kinase domain look healthy with normal blood vessel formation [9]. During the healing of wounds and gastric ulcers, expression of VEGF and VEGF receptors (VEGFRs) is elevated [10,11]. However, it remains unknown whether VEGFR1 signaling is essential for ischemic recovery and angiogenesis.

Bone marrow (BM)-derived cells are composed of hematopoietic stem cells (HSC) and other types of precursor cells has an important role in tissue repair and regeneration [12,13]. VEGF, assisted in part by VEGFR1, helps recruit BM-derived cells to ischemic tissue by stimulating the release of stromal-derived factor (SDF)-1 from platelets. This factor promotes the retention of BM-derived cells at damaged sites through its receptor, C-X-C chemokine receptor type 4 (CXCR4) [14]. The mobilization of cells that express VEGFR1 and CXCR4 [15] to sites of angiogenesis in ischemic tissues is critical for revascularization [14], suggesting that VEGFR1 signaling is potentially important. We have already reported that BM-derived CXCR4^+^VEGFR1^+^ induce gastric ulcer healing and tumor metastasis [10,11,16,17], but it remains unclear whether VEGFR1 signaling and the recruitment of BM cells are involved in the recovery of ischemic tissues remains to be elucidated.

We investigated the role of VEGFR1 signaling in the recovery from ischemia by using a domain-specific knockout mouse lacking the VEGFR1 intracellular tyrosine kinase domain (TK^-/-^). We also examined whether recovery was facilitated by the recruitment of BM progenitor cells expressing VEGFR1 and CXCR4. The results indicate that VEGFR1-TK signaling is key regulator mobilization of proangiogenic CXCR4^+^VEGFR1^+^ to the ischemic muscle and that the contribution of BM-derived CXCR4^+^VEGFR1^+^ depends on VEGFR1-TK signaling.

## Methods

### Animals and Surgery

Male 8-week-old C57Bl/6 mice were obtained from Crea Japan (Tokyo, Japan). VEGFR1-TK knockout mice (TK^-/-^) were developed on a C57Bl/6 hybrid background [18]. The model of hind limb ischemia has been described previously [19,20]. Briefly, a slit was made in the abdominal skin, permitting dissection to expose the femoral artery in the upper part of the left limb. The artery was ligated both proximally and distally with 6–0 silk sutures, the intervening 6 mm section was excised, and the incision was closed [19,20]. The VEGF-A neutralizing antibody (0.5 mg/kg body weight, R&D System Inc) [21], VEGFR2 tyrosine kinase inhibitor ZD6474 [22] (3 mg/mouse, Astra Zeneca) and CXCR4 antibody (clone 2B11, 30 μg/mouse, BD Biosciences) were injected daily into the mice after femoral artery ligation. Mice were anesthetized by intraperitoneal injection of ketamine (5 mg/kg body weight) and xylazine (100 mg/kg body weight) throughout the experiments, and its adequacy was monitored from the disappearance of the pedal withdrawal reflex. All animal experimental procedures were approved by the Animal Experimentation and Ethics Committee of the Kitasato University School of Medicine (2013–072), and were performed in accordance with the guidelines for animal experiments set down by Kitasato University School of Medicine and conformed to the Guide for the Care and Use of Laboratory Animals published by the US National Institutes of Health (NIH Publication No. 85–23, revised 1996). The mice were maintained at a constant humidity (60% ± 5%) and temperature (20°C ± 1°C) and were kept continuously on a 12-hour light/dark cycle. All animals were provided with food and water ad libitum. At the end point of experiments, mice were sacrificed by an intraperitoneal administration of an overdose of pentobarbiturate (100 mg/kg). Mice exhibiting symptoms of infection including suppressed appetite, purulent discharge from the wound were removed from the study prior to the study endpoint.

### Laser Doppler Imaging

Blood flow to the right and left hind limbs was assessed by scanning the lower abdomen and limbs of the mice using scanning laser Doppler imaging (LDI) (Lisca PIM II, Perimed, Sweden). The ratio of blood flow in the ischemic (left) limb to that in the control (right) limb was calculated by dividing the integrated blood flow in an area that included the left foot pad by the integrated blood flow in an area of the same size that included the right foot pad. Blood flow measurements were assessed by scanning preoperatively, postoperatively, and on days 3, 7, 14, 21, and 28.

### Morphological Quantification of Blood Vessel Formation

After dissection, the muscle tissues were immediately fixed with 4% paraformaldehyde in 0.1 M PBS (pH 7.4), dehydrated in a graded series of ethanol solutions, and embedded in paraffin. Sections (4 μm) of paraffin-embedded tissues were mounted on glass slides, deparaffinized with xylene, and placed in cold (4°C) acetone for immunostaining. Staining was performed with a Vectastain ABC kit (Vector Lab, Burlingame, CA, USA) as follows: sections were 1) incubated with diluted normal horse serum, 2) incubated overnight with diluted (in X500) CD31 (or platelet-EC adhesion molecule [PECAM]) polyclonal antibody (Sigma), 3) incubated with biotinylated anti-IgG, 4) incubated with avidin-biotin-peroxidase complex, and 5) placed in 0.02% 3,3'-diaminobenzine (DAB) and 0.3% nickel ammonium sulfate in 50 mM Tris-HCl buffer (pH 7.4). Color was developed by immersion in DAB solution containing 0.005% H_2_O_2_, and examination and photomicrography were performed with a light microscope. To avoid nonspecific staining of CD31, antibody was diluted 500×. Capillary density in the ischemic muscle tissues, a parameter of angiogenesis, was assessed in a blinded manner according to previously described methods [4] with some modifications. For quantitation, the number of CD31^+^ cells was counted in 20 randomly selected transverse sections in each animal. The results were averaged, and the number of CD31-positive cells was taken as the capillary density [21].

### Flow Cytometric Analysis

Blood was collected via the tail vein on day 0 and 7 after surgery. Flow cytometric analyses were performed as described previously [23]. The cells were labeled with fluorescein isothiocyanate-labeled anti-VEGFR1 antibody and phycoerythrin-labeled anti-CXCR4 isotype control antibody (Becton Dickinson Biosciences) in the presence of the anti-FcR monoclonal antibody 2.4G2 (Becton Dickinson Biosciences). Stained cells after washing were examined on FACS Calibur flow cytometer (Becton Dickinson Biosciences) with CELL QUEST software. The WBC number was assessed by counting the number of nucleated cells using Celltac α (MEK-6450; Nihon Kohden, Tokyo, Japan). The percentage of CXCR4^+^VEGFR1^+^ cells was calculated using the flow cytometry results. The number of CXCR4^+^VEGFR1^+^ cells was estimated by multiplying the flow cytometry results by the WBC count.

### Measurement of VEGF, SDF-1, SCF and pro-MMP-9 Levels

To determine the plasma levels of VEGF, SDF-1, SCF and BM level of pro-MMP-9, plasma and BM via the femur was collected and stored at -20°C until use. Plasma levels of VEGF, SDF-1, SCF and the BM level of pro-MMP-9 were assessed by murine-specific ELISA (R&D Systems, Minneapolis, MN, USA). These experiments were performed in duplicate.

### Determination of VEGFA, VEGFR1-3, CD31, CXCR4, and SDF-1 mRNA Levels in Ischemic Tissues by Real-Time PCR

Transcripts encoding VEGFR-1, -2, -3, CD31, CXCR4, SDF-1, and β-actin were quantitated by real-time (RT)-PCR analysis. Total RNA from ischemic tissues was extracted with TRIzol reagent (Invitrogen, Carlsbad, CA, USA), and the amount of RNA was measured with a BioPhotometer (Eppendorf Co. Ltd., Tokyo, Japan). Synthesis of first-strand cDNA from total RNA was performed with 1μg of total RNA, 200 U of reverse transcriptase (ReverTra Ace, Toyobo, Osaka, Japan), 40 nmol of dNTP mixture (Toyobo), 20 U of RNase inhibitor (Toyobo), and 20 pmol of oligo(dT)_20_ in a total volume of 40μL. The reactions were incubated initially at 30°C for 10 minutes, and then at 42°C for 40 minutes, followed by inactivation at 99°C for 5 minutes. Real-time PCR primers were designed using the Primer3 software (http://primer3.sourceforge.net/) using data from GenBank. The DNA sequences of mouse primers used for real-time PCR are described in Table 1.

**Table 1:** Real-time PCR primers used in the present study.

Gene	sense	antisense
VEGFR1	GTCTCCATCAGTGGTCTACG	CCCGGTTCTTGTTGTATTTG
VEGFR2	CTGCCTACCTCACCTGTTTCC	CGGCTCTTTCGCTTACTGTT
VEGFR3	GGAAGGCTCTGAAGATAAAGG	ACAGAAGATGAGCAGGAGGAG
CD31	ACTTCTAACTCCAACSGCGA	CCATGTTCTGGGGTCTTTAT
VEGFR-A	GAGAGAGGCCAAGTCCTTT	TTGGAACCGGCATCTTTATC
SDF-1	CAGAGCCAACGTAAGCA	AGGTACTCTTGGATCCAC
CXCR4	CTCTGAAGAAGTGGGGTCTGG	AAGTAGATGGTGGGCAGGAAG
β-actin	TAGACTCGAAGCAGGAGATGG	CAAGAAGGAAGGCTGGAAAG

### Bone Marrow Transplantation

BM transplantation was performed as described previously [24]. Donor BM cells from TK^-/-^ and their WT counterparts were harvested using the same method. The BM mononuclear cells of each donor (2 × 10^6^ cells / 200 μL of PBS) were transplanted via the tail veins of irradiated WT mice.

### Immunofluorescence

Tissue samples from the ischemic hind limb and the control limb were fixed with 10% neutral buffered paraformaldehyde at 4°C for 1 hour. Following cryoprotection with 30% sucrose/0.1 M phosphate buffer (pH 7.2), 10 to 20 μm-thick cryostat sections were made. Nonspecific staining was blocked by incubation with 1% bovine serum albumin (BSA)/PBS for 1 hour. The sections were incubated with anti-mouse CXCR4 antibody (1:200; Life Span Biosciences, Seattle, WA), anti-mouse VEGFR1 (1:200; Santa Cruz Biotechnology, Dallas, TX, USA), and anti-goat CD31 (1:100; Santa Cruz Biotechnology) in 1% BSA/PBS at room temperature for 1 hour or overnight. The sections were washed three times in PBS and treated with secondary antibodies, including Alexa Fluor 488-conjugated anti-goat IgG (Molecular Probes Inc.) and Alexa Fluor 594-conjugated anti-rabbit IgG (Molecular Probes Inc.). The combinations of primary antibodies were as follows: 1) rat anti-mouse CXCR4 antibody and rabbit anti-mouse VEGFR1, 2) rat anti-mouse CXCR4 antibody, rabbit anti-mouse VEGFR1 and goat anti- rabbit CD31. Labeled sections were observed using confocal laser scanning microscopy (LSM710; Carl Zeiss Micro Imaging GmbH., Oberkochen, Germany). Serial optical sections, collected at 1 μm intervals along the z-axis, were overlaid into the final images using the ZEN-2008 software package (Carl Zeiss MicroImaging GmbH.) installed on the LSM710.

### Analysis of *In Vivo* Microscopy

Vessels at the same optical location on the surface of the hind limb muscle tissue were analyzed in 10 perifemoral or muscular regions in each animal [25]. The total length of microvessels on which rhodamine 6G-labeled platelets were deposited per observation area was measured. The results were averaged, and the data were expressed as the density of microvessels.

### Statistics

Data are expressed as the means ± standard deviation (SD). All statistical analyses were performed using GraphPad Prism version 5.01 (GraphPad Software, La Jolla, CA). Statistical comparison between two groups were used the student’s t-test. Comparisons more than two groups were analyzed using one-way ANOVA. Comparisons the time point effects were analyzed by repeated-measures ANOVA. If applicable with a repeated measure approach, Bonferroni post hoc test was performed. A P-value of less than 0.05 was considered statistically significant.

## Results

### The expression of VEGFR1 increases after femoral artery ligation

**Fig 1:**
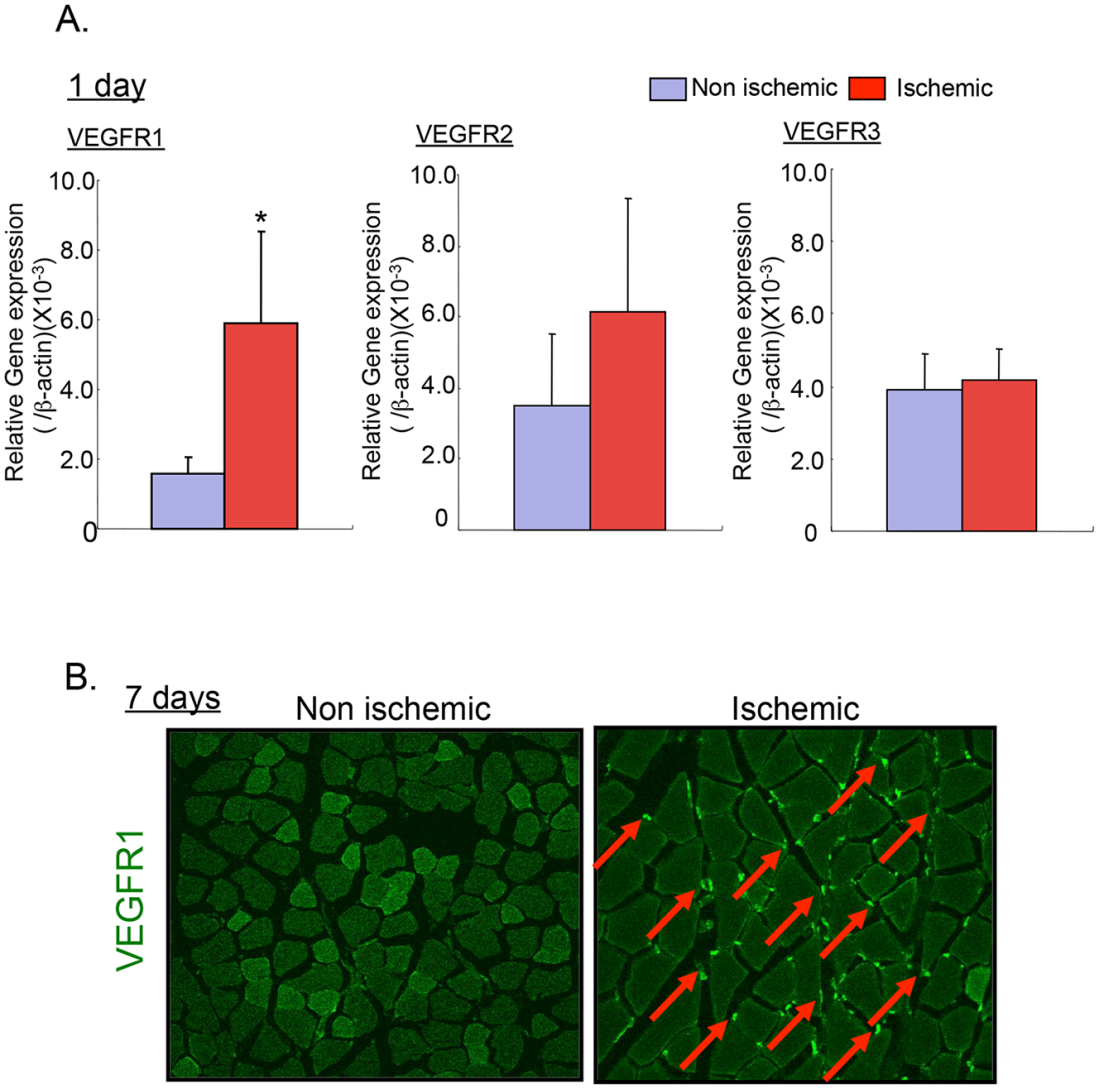
The expression of VEGFR1 was enhanced in ischemia muscle. (A) The expression of VEGFRs on day 1 following femoral artery ligation. Data aremeans ± SD from n = 5 mice/group. *P<0.05 versus control mice. (B) The accumulation of VEGFR1^+^ cells on day 7 increased during the ischemic condition.

VEGF and its receptor (VEGFR), including VEGFR1, VEGFR2, and VEGFR3, are essential for angiogenesis under physiological and pathological conditions. VEGF-A is one of the most potent angiogenesis stimulators, and binds two tyrosine kinase receptors, VEGFR1 and VEGFR2. VEGFR3 is a receptor for VEGF-C and VEGF-D. To determine which of the VEGF receptors, we measured the mRNA levels of VEGFR1–3 in muscle by real-time PCR. On day 1 after femoral ligation, under ischemic condition, the expression of VEGFR1 was more than 2-fold higher than the expression of the other VEGFRs (Fig 1A). Moreover, immunofluorescence analysis showed that the abundance of VEGFR1-positive cells on day 7 was increased compared to that of non-ischemic muscle (Fig 1B). These results indicate that VEGFR1 is a key regulator in the recovery from ischemia.

**Fig 2:**
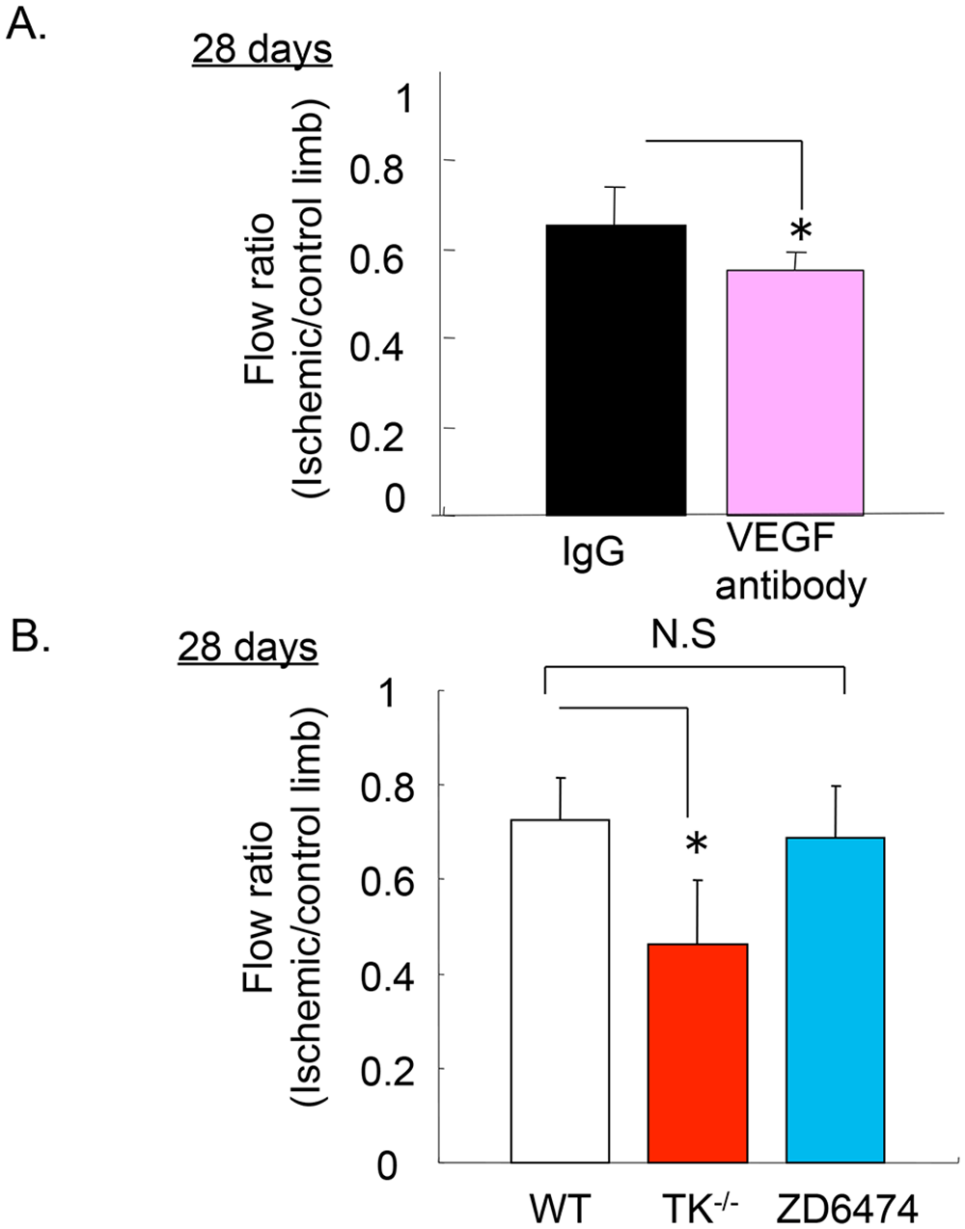
The effect of VEGFR1 in the recovery from ischemia. (A) Blood flow recovery was suppressed following VEGF-neutralizing antibody treatment on day 28 after surgical treatment. Data are means ± SD from n = 6 mice/group. *P<0.05 versus control (IgG treated mice). (B) Blood flow recovery was impaired in TK^-/-^ on day 28 after surgical treatment. Data are means ± SD from n = 8 mice/group. *P<0.05 versus WT.

Next, we confirmed whether VEGF signaling affects ischemic revascularization. We injected VEGF-A-neutralizing antibody following femoral artery ligation. Mice treated with the antibody had significantly impaired blood flow recovery (28 days: IgG control antibody: 0.65 ± 0.09, VEGF-A antibody: 0.55 ± 0.04, *P<0*.*05*, Fig 2A). We then estimated the effects of VEGFR1 and VEGFR2 on ischemic revascularization in TK^-/-^ treated with the VEGFR2-TK inhibitor, ZD6474. Treatment with ZD6474 did not affect blood flow recovery when compared to treatment with the vehicle (28 days: vehicle: 0.72 ± 0.091, ZD6474: 0.69 ± 0.11, P = 0.49). By contrast, blood flow recovery was significantly impaired in TK^-/-^ (28 days: 0.46 ± 0.14, *P<0*.*05*).

### Inhibition of VEGFR1-TK signaling impairs ischemic recovery and the healing of ischemic muscle

**Fig 3:**
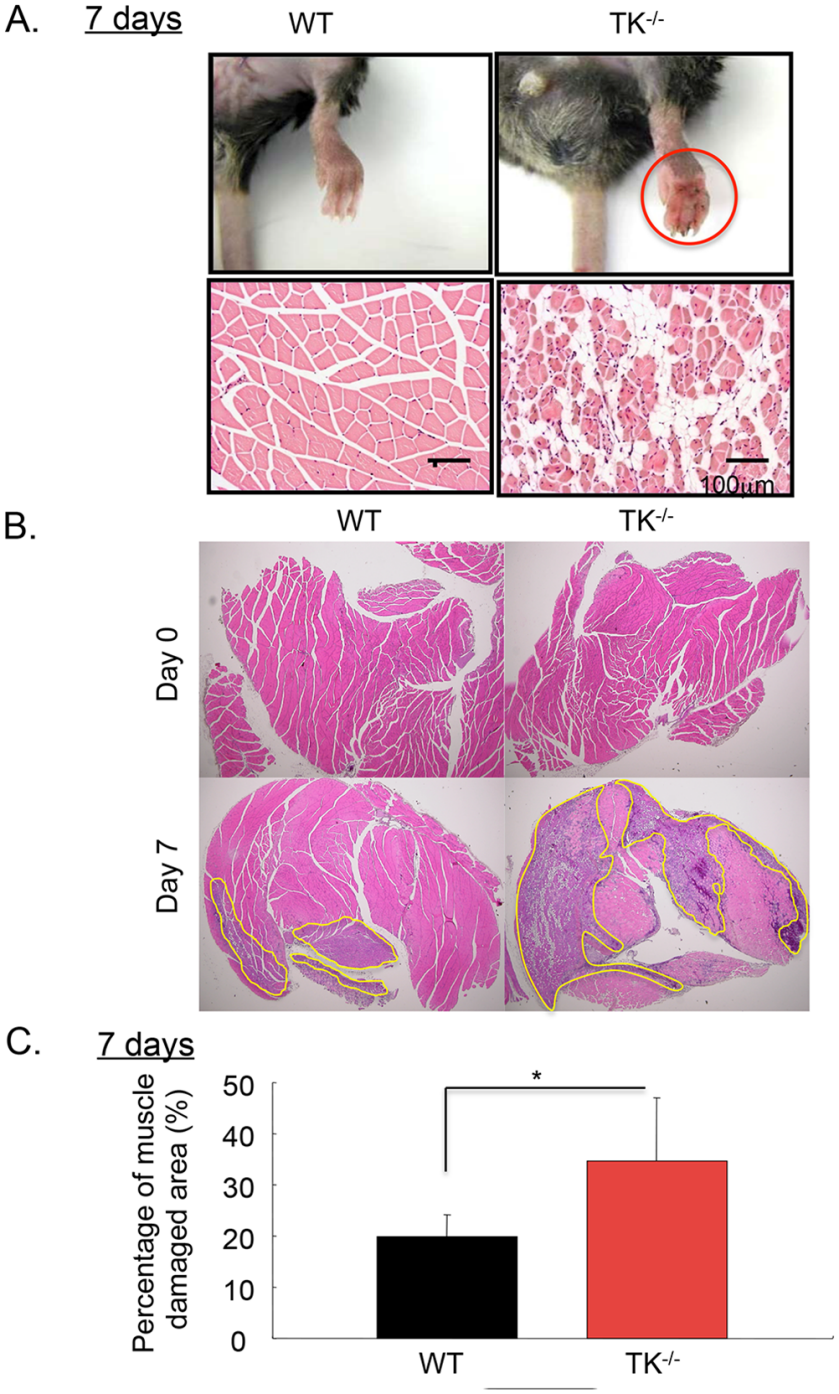
The effect of VEGFR1-TK signaling on the healing of ischemic muscle. (A) Typical appearance of ischemic footpad (upper pannel) and muscle (lower panel) Bar = 100 μm. (B) Muscle damaged area was decreased in WT compared to TK^-/-^ on day 7 after surgical treatment (upper panel). Percentage of muscle damage area was significantly suppressed in WT compared to TK^-/-^. Bar = 100 μm yellow circle area indicates damaged area. Data are means ± SD from n = 10 mice/group. *P<0.05 versus control (WT).

As shown in Fig 3A, TK^-/-^ had skin ulcers and swelling on their right footpads on day 7 (upper panel). Angiogenesis is also critical for healing muscle after ischemia. The loss of skeletal myocytes, which were substituted by fat, occurred in TK^-/-^, but not in WT (lower panel). At 7 days after femoral artery ligation, the muscle-damage area, including necrosis and fat degeneration (Fig 3B), was significantly larger in TK^-/-^ than in WT (WT: 19.8 ± 4.3%, TK^-/-^: 34.6 ± 12.4%, *P* < 0.05, Fig 3C). This result suggests that impaired post-ischemic muscle recovery in TK^-/-^ was caused, at least in part, by inhibition of revascularization.

### Inhibition of VEGFR1-TK signaling suppresses vascular formation in ischemic muscle

**Fig 4:**
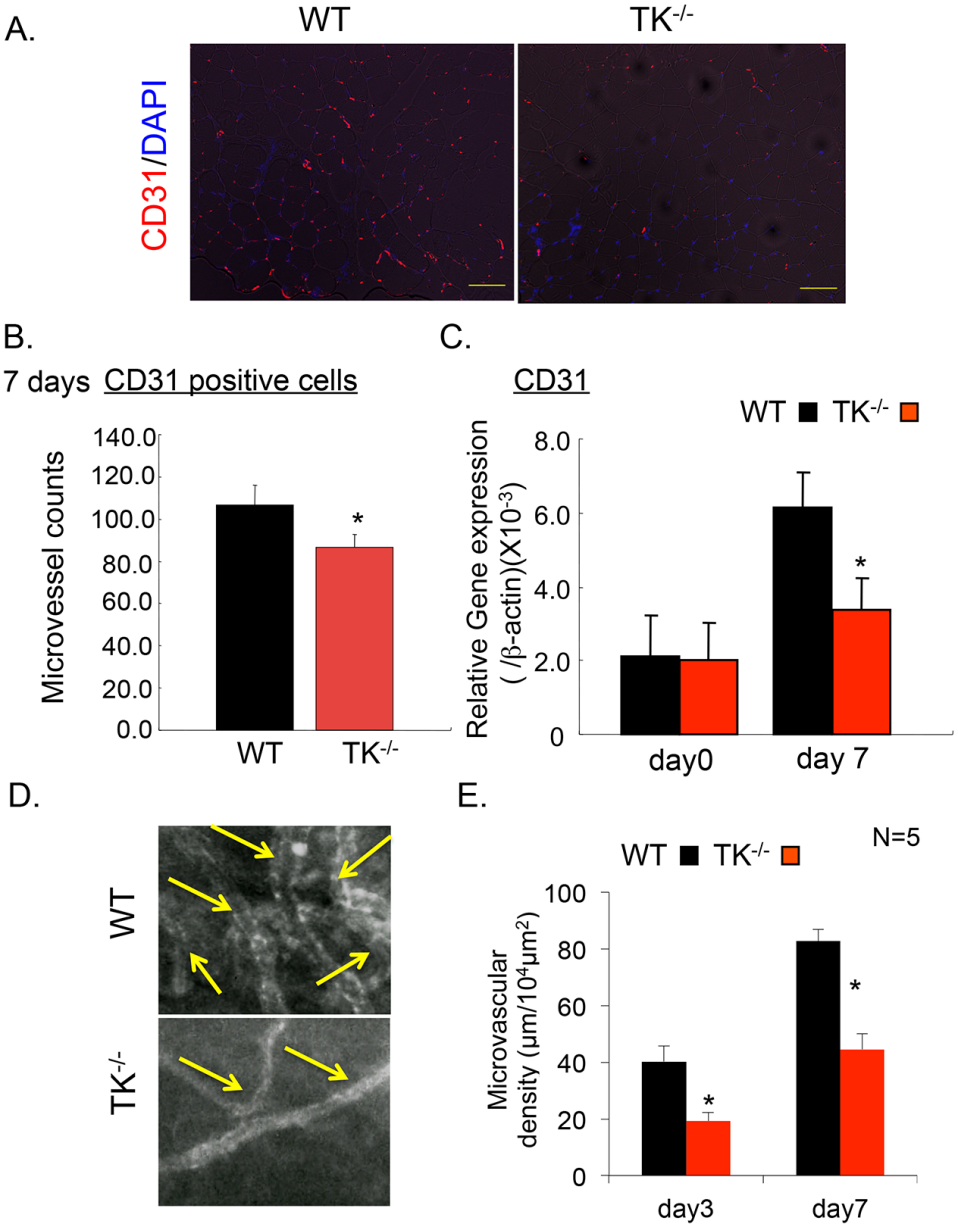
VEGFR1-TK signaling induces ischemic neovascularization. (A) The CD31 staining in ischemic muscle at day 14 in WT and TK^-/-^. Bar = 100 μm.(B) The microvessel counts were diminished in TK^-/-^ on day 7 after surgery. Data are means ± SD from n = 10 mice/group. *P<0.05 versus control (WT). (C) The expression of CD31 in ischemic muscle was suppressed in TK^-/-^ on days 7 following femoral artery ligation. Data are means ± SD from n = 6 mice/group. *P<0.05 versus control (WT). (D) *In vivo* microscopic observation at day 7 in WT and TK^-/-^. Yellow arrows indicate the newly formed vessels.(E) Quantification of microvascular density in perifemoral muscle tissues on days 3 and 7 after surgery. Data are means ± SD from n = 5 mice/group. *P<0.05 versus control (WT).

We followed the formation of the vascular network using immunohistochemical analysis and *in vivo* microscopy. In ischemic muscle tissue, the abundance of CD31-positive cells (Fig 4A and 4B), a marker for endothelial cells, and the level of CD31 mRNA (Fig 4C) were both lower in TK^-/-^ than in WT (CD31-positive cells: WT: 106.6 ± 9.5, TK^/-^: 86.5 ± 6.1, *P* < 0.05, Fig 4C). Intravital microscopy revealed that the microvascular density in the vasculature of the perifemoral site was significantly lower in TK^-/-^ on days 3 and 7 (Day 3: WT: 40.22 ± 5.50, TK^-/-^: 19.2 ± 2.95; Day 7: WT: 82.67 ± 4.15, TK^-/-^: 44.5 ± 5.58, *P* < 0.05, Fig 4D and 4E). Taken together, these findings suggest that VEGFR1-TK signaling induces angiogenesis and supports post-ischemic muscle recovery.

### The role of VEGFR1 and CXCR4 signaling in the recovery from ischemia

**Fig 5:**
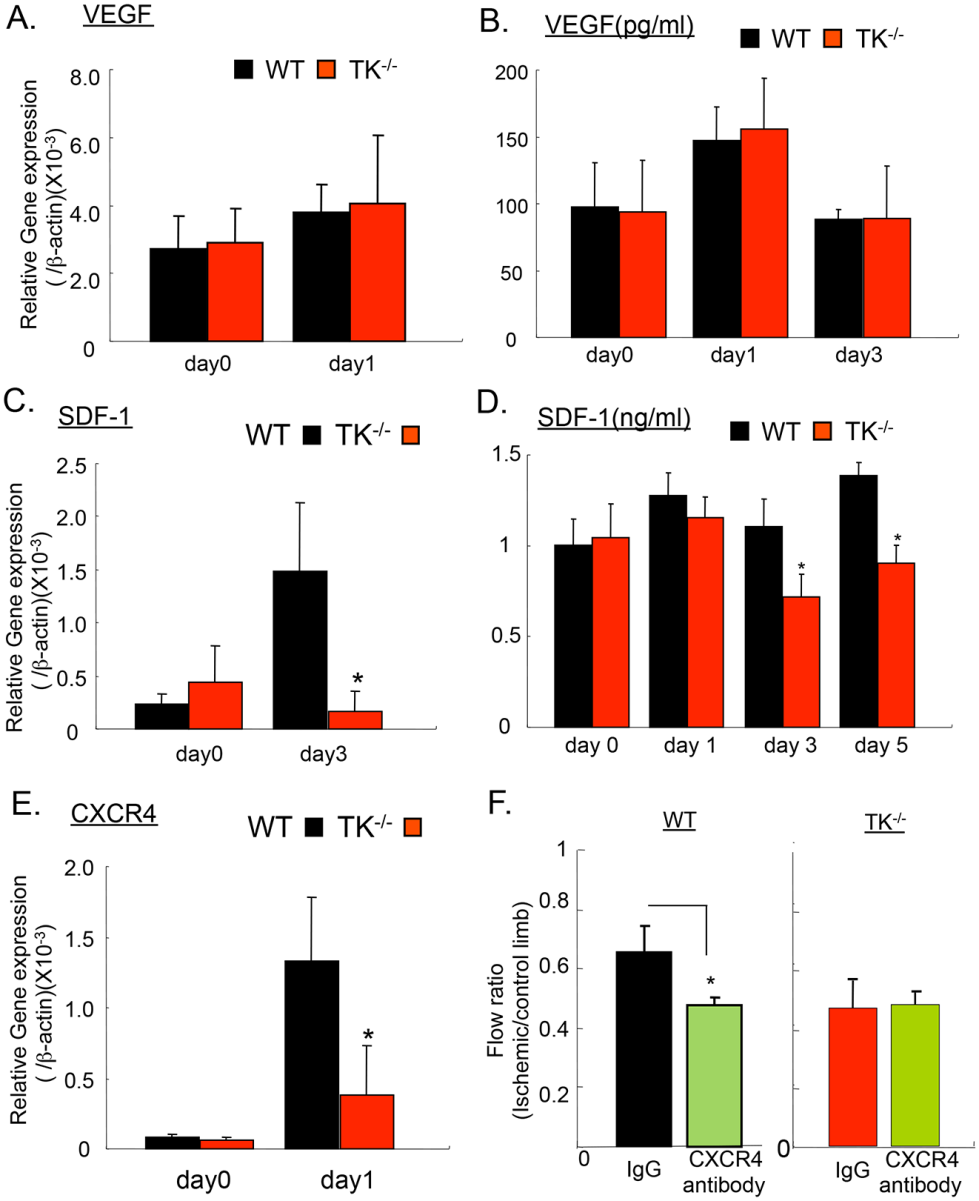
Role of VEGF and SDF-1 signaling in recovery from ischemic condition. (A) The expression of VEGF in ischemic muscle on days 0 and 1 after surgery. Data are means ± SD from n = 4 mice/group. *P<0.05 versus control (WT).(B) The concentration of VEGF in peripheral blood on day 0 and day 1 after surgery. Data are means ± SD from n = 4–5 mice/group. *P<0.05 versus control (WT).(C) The expression of SDF-1 in ischemic muscle on day 0 and day 3 after surgery. Data are means ± SD from n = 4 mice/group. *P<0.05 versus control (WT).(D) The concentration of SDF-1 in peripheral blood on days 0, 1, 3, and 5 after surgery. Data are means ± SD from n = 4–5 mice/group. *P<0.05 versus control (WT).(E) The expression of CXCR4 in ischemic muscle on day 0 and 1 after surgery. Data are means ± SD from n = 4 mice/group. *P<0.05 versus control (WT).(F) Ischemic reperfusion was suppressed by CXCR4 antibody treatment in WT, but not in TK^-/-^ on day 28 after surgical treatment. Data are means ± SD from n = 6 mice/group. *P<0.05 versus control (IgG).

The growth of granulation tissue and the generation of new microvessels through angiogenesis are stimulated by angiogenesis-stimulating factors, VEGF-A and SDF-1. VEGF and SDF-1 mobilize BM-derived VEGFR1^+^ to promote tumor growth [13,14]. The mRNA level of VEGFA (Fig 5A) and the plasma level of VEGF in both WT and TK^-/-^ were significantly elevated on day 1 relative to day 0; however, there was no difference between the groups on day 1 (147.04 ± 25.2 pg/mL vs. 155.75 ± 38.06 pg/mL, respectively; *P* = 0.716, n = 5 per group, Fig 5B). By contrast, the mRNA level of SDF-1 (Fig 5C) and the plasma level of SDF-1 were significantly lower in TK^-/-^ than in WT on day 3 (0.72 ± 0.12 ng/mL vs. 1.10 ± 0.15 ng/mL, respectively; *P*<0.05, n = 5 per group) and day 5 (0.90 ± 0.10 ng/mL vs. 1.39 ± 0.07 ng/mL, respectively; *P*<0.05, n = 5 per group, Fig 5D). Moreover, the expression of CXCR4, the specific ligand for SDF-1, was significantly suppressed in TK^-/-^ (Fig 5E).

To verify the functional role of SDF-1 in blood flow recovery, mice were treated with neutralizing antibodies against CXCR4. Treatment of WT with anti-CXCR4 antibodies impaired blood flow recovery more than treatment with the control IgG antibody (28 days: control IgG: 0.65 ± 0.11, CXCR4 antibody: 0.38 ± 0.14, n = 6 per group, *P* < 0.05, Fig 5F). In TK^-/-^, there was no difference between treatment with IgG and anti-CXCR4 antibody (28 days: control IgG: 0.46 ± 0.09, CXCR4 antibody: 0.47 ± 0.04, P = 0.84, Fig 5F). These results suggest that CXCR4 signaling promotes ischemic recovery and that CXCR4-mediated ischemic recovery is dependent on VEGFR1. Thus, both VEGFR1- and CXCR4-mediated signaling pathways are critical for blood flow recovery.

### VEGFR1-TK signaling mobilizes CXCR4^+^VEGFR1^+^ hematopoietic cells into ischemic muscle

**Fig 6:**
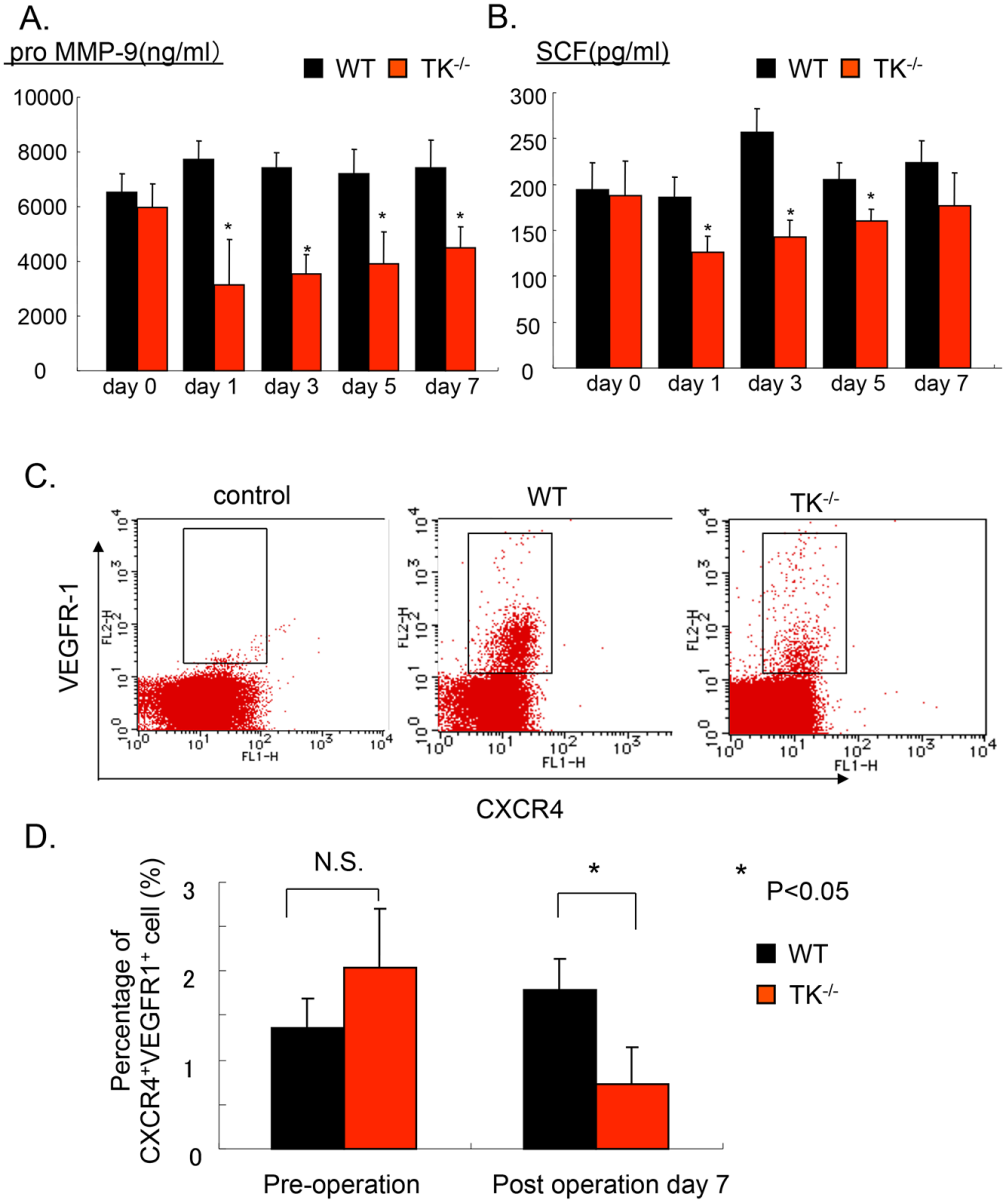
The effect of VEGFR1TK signaling on hematopoietic cytokine levels and the mobilization of CXCR4^+^VEGFR1^+^ cells. Bone marrow level of pro-MMP-9 level (A) and plasma level of SCF (B) on days 0, 1, 3, 5, and 7 after surgery. Data are means ± SD from n = 4 mice/group. *P<0.05 versus control (WT). (C) Mobilization of CXCR4^+^VEGFR1^+^ cells in peripheral blood was impaired in TK^-/-^, as compared to WT, on day 7 after surgery, as revealed by two-color flow cytometry. (D) Quantification of CXCR4^+^VEGFR1^+^ cells in peripheral blood on day 7 after surgery. Data are means ± SD from n = 4–5 mice/group. *P<0.05 versus control (WT).

**Fig 7:**
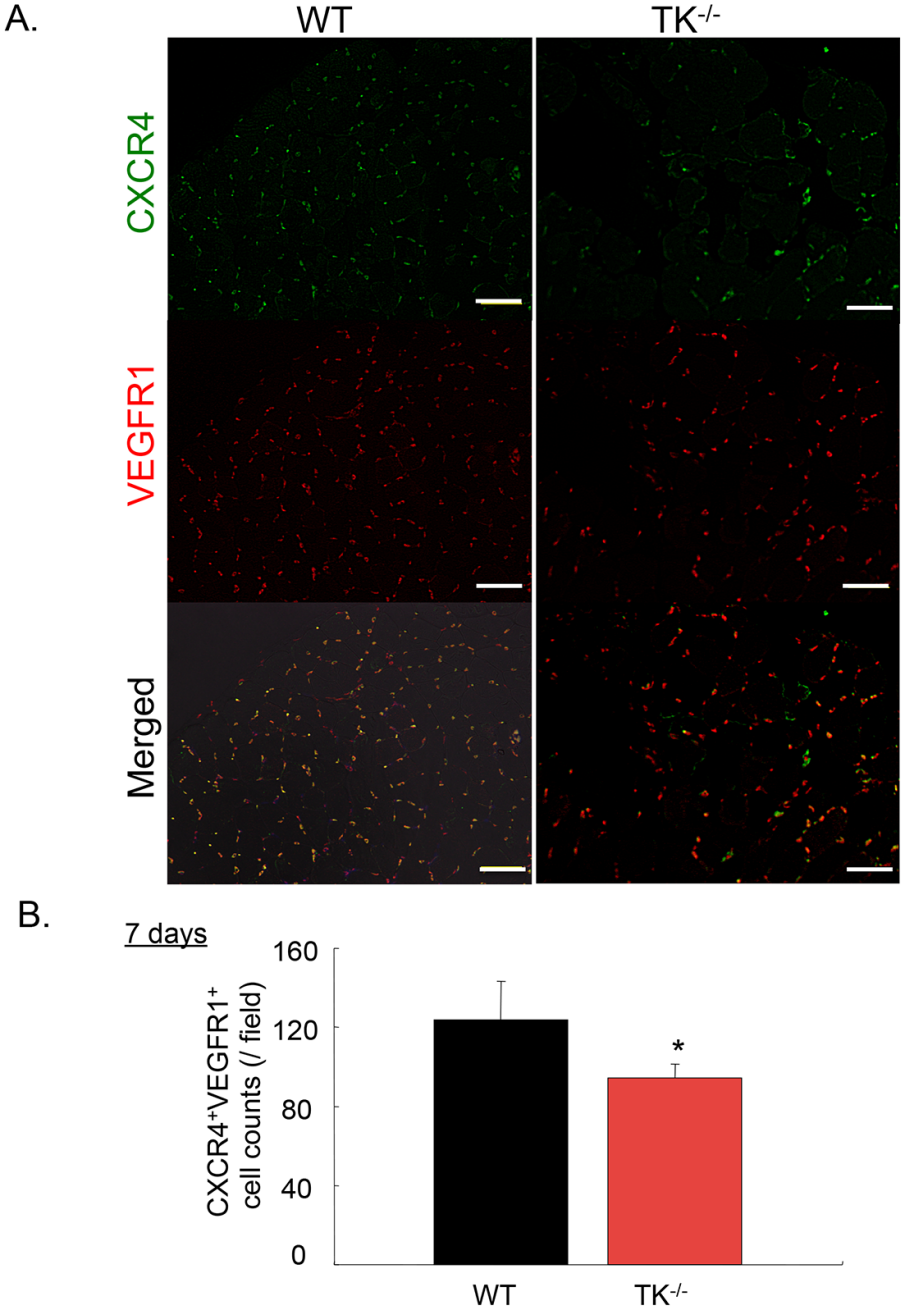
The accumulation of CXCR4^+^VEGFR1^+^ cells in ischemic muscle was diminished in TK^-/-^ mice. (A) The accumulation of CXCR4^+^VEGFR1^+^ cells was diminished in TK^-/-^ compared to WT on day 7 after surgery. Red: VEGFR1^+^ cells; green: CXCR4^+^ cells. Bar = 100 μm. (B) Quantification of CXCR4^+^VEGFR1^+^ cells in ischemic muscle on day 7 after surgery. Data are means ± SD from n = 8 mice/group. *P<0.05 versus control (WT).

BM-derived HSCs plays a crucial role in ischemic revascularization [13]. Fig 6A and 6B shows the bone marrow (BM) and plasma levels of hematopoietic cytokines, including pro-MMP-9 and stem cell factor (SCF). BM levels of pro-MMP-9 (Fig 6A) and plasma levels of SCF (Fig 6B) were significantly decreased in TK^-/-^ compared to those of WT on days 1, 3, and 5 (*P<0*.*05*). We previously reported that BM-derived CXCR4^+^VEGFR1^+^ to gastric ulcer lesions is important for ulcer regeneration [10]. Therefore, we measured the mobilization of CXCR4^+^VEGFR1^+^ cells into ischemic muscle tissues using fluorescence-activated cell-sorting analysis (FACS). On day 7, the percentage of CXCR4^+^VEGFR1^+^ cells in circulating cells was reduced in TK^-/-^ (WT: 1.87 ± 0.31, TK^-/-^: 0.63 ± 0.12, *P<0*.*05*, Fig 6C). Compared to WT, the total number of CXCR4^+^VEGFR1^+^ cells in the ischemic muscle was significantly reduced in TK^-/-^ (WT: 123.8 ± 19.51 cells/field, TK^-/-^: 94.4 ± 6.8 cells/field, *P<0*.*05*, n = 5 per group, *P<0*.*05*, Fig 7A and 7B). These findings suggest that VEGFR1-TK signaling helps promote CXCR4^+^VEGFR1^+^ mobilization into circulation and ischemic tissue.

### Transplantation of BM cells from TK^-/-^ to WT impairs blood flow recovery

**Fig 8:**
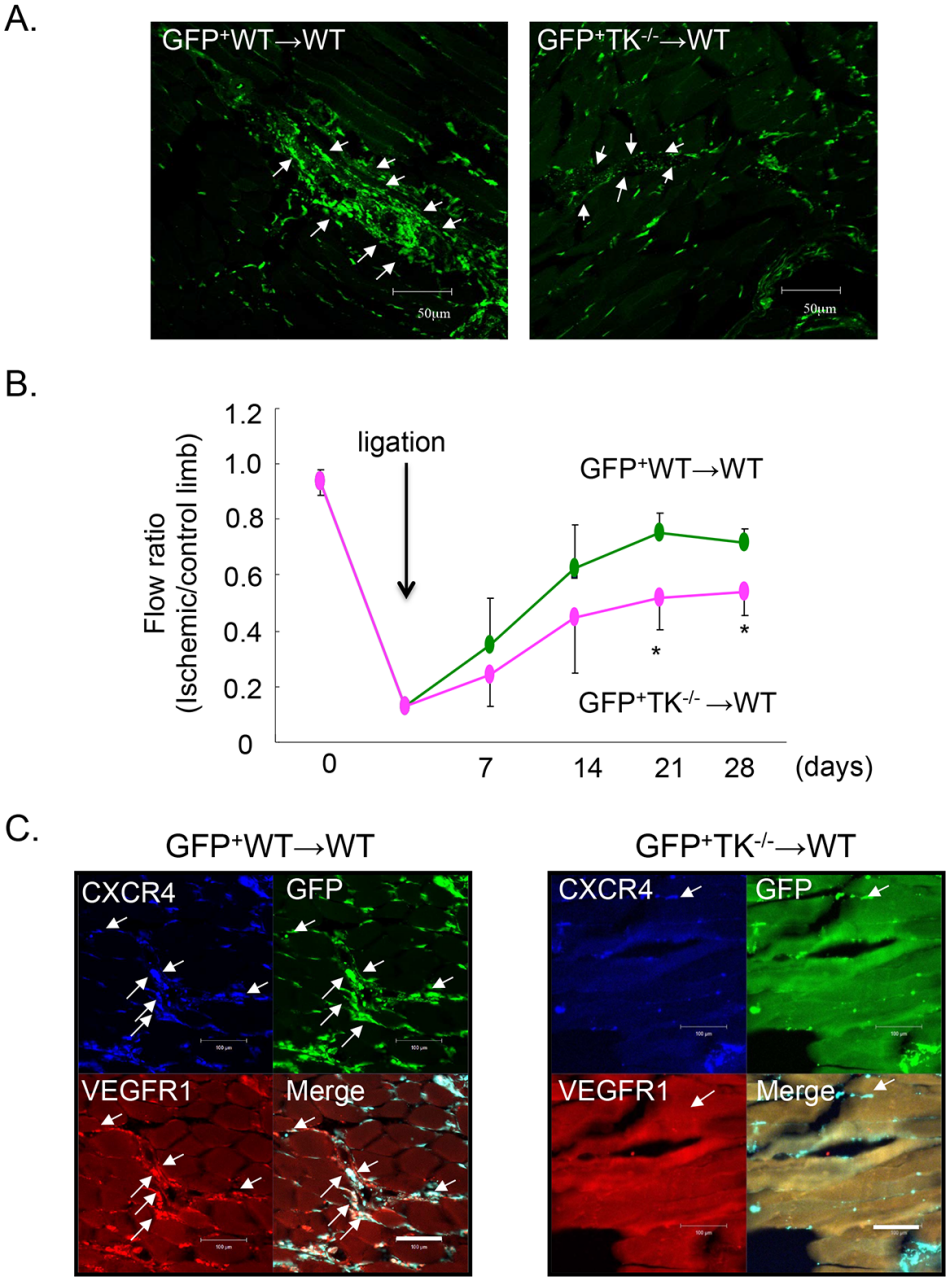
Effect of VEGFR1-TK signaling on ischemic revascularization following BM transplantation. (A) The accumulation of GFP^+^ BM cells diminished in WT transplanted with GFP^+^ TK^-/-^ BM. White arrows indicate BM-derived cells. Bar = 50 μm. (B) Blood flow recovery from the ischemic condition after transplantation of BM from WT or TK^-/-^ to WT. Data are means ± SD from n = 5 mice/group. *P<0.05 versus WT-WT transplantation. (C) The accumulation of BM-derived CXCR4^+^VEGFR1^+^ cells in ischemic muscle. Bar = 100 μm.

BM-derived HSCs is reported to play a crucial role in ischemic revascularization. To examine the contribution of BM-derived cells expressing VEGFR1-TK to angiogenesis, we selectively inhibited VEGFR1-TK signaling in BM cells by transplanting BM cells from TK^-/-^ into WT. At 28 days, blood flow recovery was reduced in GFP^+^TK^-/-^ BM-transplanted WT, compared to that in GFP^+^WT BM-transplanted WT (28 days: GFP^+^WT-WT: 0.715 ± 0.05, GFP^+^TK^-/-^ WT: 0.542 ± 0.09, *P* < 0.05, Fig 8A). Next, we confirmed whether GFP^+^ BM derived cells accumulated in ischemic tissue. GFP^+^TK^-/-^ BM-transplanted WT mice had less accumulation of GFP^+^ BM cells in ischemic muscle than did the GFP^+^WT BM-transplanted WT (Fig 8B). GFP^+^ BM-derived cells also co-stained with CXCR4 and VEGFR1 in GFP^+^WT BM-transplanted WT, but rarely co-stained in GFP^+^TK^-/-^ BM-transplanted WT (Fig 8C). These results suggest that the recruitment of BM cells expressing CXCR4 and VEGFR1 contributes to ischemic revascularization and that it depends on VEGFR1-TK signaling.

## Discussion

The objective of the present study was to reveal the role of VEGFR1-TK signaling in the blood recovery from ischemia. The results indicate that endogenous VEGFR1-TK signaling facilitates angiogenesis by recruiting BM-derived cells expressing CXCR4 and VEGFR1 to ischemic tissues. Transplantation of BM isolated from TK^-/-^ into WT demonstrated that BM-derived CXCR4^+^ VEGFR1^+^ cells diminished blood flow recovery, indicating that recovery is dependent on VEGFR1-TK signaling.

VEGF is an important factor in the normal vascular development of organ systems [26]. VEGFR1 and VEGFR2 are highly expressed in endothelial cells and lung tissue. Heterozygous VEGF^+/-^ mice die *in utero*, and post-natal inactivation of VEGF increases mortality. VEGFR2-null mice die in the embryonic stage because of a lack of blood vessels, indicating that VEGFR2 signaling is essential for the development of vascular systems. VEGFR1^-/-^ is embryonic lethal due to the overgrowth of endothelial cells and blood vessel dysfunction [27]. TK^-/-^ lack only the signaling mediated by VEGFR1 and are useful for elucidating the importance of this signaling under physiological conditions. Indeed, in our study, the plasma and mRNA levels of VEGF were equal between WT and TK^-/-^. It was already reported that TK^-/-^ reduced angiogenesis, in parallel with decreasing recruitment of VEGFR1-expressing macrophages [28].

Previous study showed that VEGFR1 levels in ischemic muscle were enhanced compare to non-ischemic muscle [29]. In the current study we showed the expression of mRNA level of VEGFR-1 increased compared to other receptors, VEGFR2 and VEGFR3, in ischemic muscle. This result focused our investigations onto VEGFR1’s role in the recovery from ischemia. The recovery was not affected by inhibition of VEGFR2-TK (ZD6474), but was reduced significantly by the knockout of VEGFR1-TK. The expression of CD31 in ischemic muscle and the microvascular density were both significantly reduced in TK^-/-^. Moreover, *in vivo* intravital microscopy analysis revealed that the microvascular density was diminished in TK^-/-^. In addition, the muscle-damage area was significantly larger than in WT. These results suggest that VEGFR1-TK signaling is a key regulator in recovering from ischemia and damaged tissue.

In the current study, we had used ZD6474 as a specific inhibitor of VEGFR2 [22]. ZD6474 treatment failed to recover the blood flow form ischemic condition, suggesting that VEGFR2 plays a minor role in revascularization after hind limb ischemia. However, recent reports suggest that ZD6474 inhibit VEGFR1 signaling in human cancer cell lines [30]. A kinase assay also revealed that ZD6474 efficiently inhibit not only VEGFR2 (IC_50_, 38 nmol/L) but also VEGFR1 (IC_50_, 150 nmol/L) [30], indicating that ZD6474 displays a broader inhibitory activity than reported previously [22]. Based on these observations, ZD6474 has the possibility to inhibit signaling both from VEGFR2 as well as VEGFR1. Further studies are needed to elucidate the role of VEGFR2 signaling in ischemia-induced angiogenesis using specific inhibitors of VEGFR2.

Two types of VEGFR1 receptors have been characterized; one for a full-length VEGFR1 receptor, and another for a ligand-binding region as a soluble form of the VEGFR1 protein [5,9]. In the present study, TK^-/-^, which lacks TK domain of VEGFR1, exhibits impaired angiogenesis after hind limb ischemia, indicating that VEGFR1 signaling is essential for ischemia-related revascularization. On the other hand, Murdoch et al [31] showed that plasma levels of s-VEGFR1 was increased after femoral artery ligation using glutaredoxin-1 transgenic mice and showed that the recovery from ischemic condition was delayed in these mice. These results indicate that the induction of s-VEGFR1 is relevant to suppressed blood flow recovery from hind limb ischemia. This soluble VEGFR1 could trap VEGF-A to prevent its binding to VEGFRs. These also suggest that s-VEGFR1 inhibits VEGF activity without affecting intracellular signaling pathway of VEGFRs. Therefore, VEGFR1 acts as both a negative regulation of angiogenesis through its extracellular domain and a positive regulation of angiogenesis through its TK. We further need to elucidate the relative importance of the expression of s-VEGFR1 between WT and TK^-/-^ during hind limb ischemia.

Stromal cell derived factor-1 (SDF-1) is a chemokine that participates in the regulation of tissue homeostasis [32], immune surveillance [33], inflammatory responses, and cancer development [34, 35]. It has been already reported that SDF-1 /CXCR4 axis related to chemotaxis, cell survival, angiogenesis, metastasis formation, and gene transcription [36, 37]. SDF-1/CXCR4-induced gastric ulcer healing depends on VEGFR1-TK signaling [11]. In this study, the plasma and mRNA levels of SDF-1 were reduced in TK^-/-^. Furthermore, the expression of CXCR4, the specific receptor of SDF-1, was also diminished in TK^-/-^ ischemic muscle. In WT, blood flow recovery was suppressed by injection with CXCR4 antibody, but not in TK^-/-^. These results indicate that SDF-1/CXCR4 signaling also contributes to blood flow recovery and depends on VEGFR1-TK signaling.

BM-derived cells have the potential to differentiate into the cellular components of vasculature and to increase the perfusion of the ischemic organ. VEGF and SDF-1 are not only angiogenesis stimulators, but also mobilizers of BM stem cells [38, 39]. TK^-/-^ with an op/op genetic background has significant suppression of BM reconstruction, indicating that VEGFR1 signaling is partly involved in BM formation [40]. BM-derived cells expressing VEGFR1 induce post-natal vasculogenesis [41] and tumor growth and metastasis formation [42]. In this study, blood flow recovery was impaired in TK^-/-^ compared to that in WT.

The involvement of BM-derived cells critically depends on MMP-9. BM-derived circulating endothelial progenitors (CEP) reside in a quiescent state until a microenvironment switch, which depends on the up-regulation of MMP-9 by BM cells, signals them to differentiate and mobilize. This switch is caused by the increased bioavailability of SCF, released by stress, which supports hematopoiesis and angiogenesis [43]. We previously demonstrated, by using the ischemic hind limb model, that MMP-9 attenuates the remobilization of hemangioblasts expressing CXCR4^+^VEGFR1^+^ from BM [14]. In this study, the hematopoietic stimulating factors SCF and pro-MMP-9 were suppressed in TK^-/-^. These results indicate that VEGFR1-TK signaling induces the mobilization of BM cells and led us to ask whether bone marrow-derived stem cells expressing VEGFR1 and CXCR4 have a critical role in the recovery from ischemia. The number of CXCR4^+^VEGFR1^+^ cells in the peripheral blood and the accumulation of these cells in ischemic muscle was diminished in TK^-/-^. These results suggest that the recovery from ischemia is dependent on the accumulation of CXCR4^+^VEGFR1^+^ cells.

As mentioned above, BM-derived cells assist in the recovery of damaged tissue. Impaired mobilization and recruitment of BM-derived cells are responsible for delayed cardiovascular tissue repair in patients with aging and diabetes [44–46]. Therefore, we examined the effect of the accumulation of BM-derived CXCR4^+^VEGFR1^+^ cells on revascularization through a bone marrow transplantation experiment. The accumulation of GFP^+^ BM cells in ischemic muscle was diminished in the mice with GFP^+^TK^-/-^ BM compared to those with GFP^+^WT BM. The recovery from ischemia was also suppressed. Moreover, these accumulated GFP^+^ BM cells co-stained with CXCR4 and VEGFR1 in mice transplanted with GFP^+^WT BM, but rarely in those with GFP^+^TK^-/-^ BM. These results suggest that VEGFR1-TK signaling induces the mobilization of CXCR4^+^VEGFR1^+^ cells from BM and induces recovery from ischemia. However, Grochot-Przeczek et al [47] reported that a potent mobilization of BM-derived cells into the circulation during hind limb ischemia did not promote diabetic tissue revascularization. This suggests that mobilization of BM-derived cells is not always associated with revascularization of ischemic tissues in certain pathological conditions.

VEGFR1 is highly expressed in monocytes and macrophages at both the mRNA and protein levels [18, 48], and VEGFR1 is important for VEGFA-dependent migration of these cells [49]. VEGFR1^+^ macrophages, co-staining with F4/80 and CD11b, also accumulate in the liver during the repair phase of ischemic/reperfusion injury [50]. Furthermore, VEGFR1^+^ cells and CXCR4^+^ cells are positive for CD11b, a marker for macrophages, indicating that CXCR4^+^VEGFR1^+^ cells appear to be originating from monocytes or macrophages and are crucial for angiogenesis [11].

MMPs are reported to play a critical role in inflammation, tissue repair, and tumor invasion. Hiratsuka et al [18] showed that MMP-9 expression was significantly increased in the pre-metastatic lung of WT after tumor implantation compared to TK^-/-^. This result indicates that up-regulation of MMP-9 expression depends on VEGFR1TK signaling. MMP-9 also plays an important role in ischemia-induced revascularization through the recruitment of macrophages [14,51]. We previously reported that the accumulation of macrophages was significantly diminished in TK^-/-^ during the process of tissue repair in the models of gastric ulcer [11] and hepatic ischemia/reperfusion [50]. These findings indicate that VEGFR1 signaling plays a critical role in the recruitment of macrophages into the tissue through MMP-9 activity.

VEGFR2 activates a number of kinases that phosphorylate downstream signal transduction proteins including phospholipase (PLC), Src and phosphoinositide 3-kinase (PI3K). Di et al [52] have shown that VEGFR2 signaling is involved in hypoxia-induced apoptosis in endothelial cells (ECs) and endothelial progenitor cells (EPCs), which is associated with attenuated activation of Src and PI3K/Ark. Because signaling pathways of Src and PI3K/Ark also appear to be important for VEGFR1 activation [53], inhibition of activation of Src and PI3K/Ark signaling pathways in TK^-/-^ would contribute to apoptosis in ischemic muscle and CXCR4^+^VEGFR1^+^ cells, leading to impaired angiogenesis in response to hind limb ischemia. Further experiments are needed to explore the involvement of VEGFR1 in apoptosis in ECs during hind limb ischemia.

Our results indicate that VEGFR1 activation promotes angiogenesis in response to hind limb ischemia. In this regard, selective VEGFR1 agonist, placenta growth factor (PlGF), would be useful to facilitate revascularization during hind limb ischemia. Indeed, PlGF treatment was reported to enhance the revascularization of ischemic tissues including the heart and hind limb [54]. These results supported that the selective VEGFR1 agonist or PlGF might serve as a potential novel therapeutic tool for improvement with angiogenesis in ischemic tissue.

In conclusion, our study indicates that VEGFR1-TK signaling plays a critical role in the recovery from ischemia by being involved in the mobilization of hematopoietic cells expressing CXCR4^+^VEGFR1^+^ from BM. Moreover, a highly selective VEGFR1 agonist or PlGF might be useful for treating peripheral artery disease.
